# School-based intervention for anxiety using group cognitive behavior therapy in Pakistan: a feasibility randomized controlled trial

**DOI:** 10.1186/s41155-024-00311-4

**Published:** 2024-08-19

**Authors:** Saman Ijaz, Iffat Rohail, Shahid Irfan

**Affiliations:** 1 Clinical Psychology, Public Sector Organization, Islamabad, Pakistan; 2 Department of Psychology, Foundation University, Islamabad, Pakistan; 3 Afghan Migrants and Host Communities, International Organization for Migration, Islamabad, Pakistan

**Keywords:** CBT, Pakistan, Anxiety, School children, Randomized controlled trial, Feasibility

## Abstract

**Background:**

Anxiety and mood disorders are the main cause of illness in people under the age of 25, accounting for 45% of the global disease burden, whereas 4.6% of teenagers aged 15 to 19 are predicted to experience anxiety. Pakistan country, with a population of 200 million, has the worst mental health indicators and fewer than 500 psychiatrists. Despite the existence of various treatments for anxiety, this goes unrecognized and untreated. Due to a lack of awareness, evaluation, prevention, and interventional programs related to being implemented among adolescents in Pakistan, there is a rise in mental health issues in the earlier years of life. It calls for a critical need for indigenous, evidence-based interventions. The present study aimed to evaluate the feasibility of cognitive behavioral therapy (CBT)-based interventions to reduce anxiety symptoms among school children in Pakistan.

**Methods:**

This study was a pre-post design, two-arm, single-blinded, feasibility, randomized controlled trial. Thirty-four participants (experimental group, *n* = 17; control group, *n* = 17) were recruited from four semi-government schools in Rawalpindi with a mean age of 15 (*M* = 15, *SD* = 0.73). Two instruments Beck Anxiety Inventory for Youth (BAI-Y II) and BASC-3 Behavioural and Emotional Screening System Student (BESS-SF) were used to assess the severity of symptoms. Participants in the intervention arm received eight-group therapy CBT sessions. A two-way factorial analysis was used to examine the efficacy of CBT in reducing symptom severity.

**Results:**

This study’s findings showed that in comparison to the wait-list control group, CBT successfully improved anxiety symptoms among school children while enhancing their social skills.

**Conclusion:**

This study will help improve the treatment for anxiety in Pakistan by prioritizing school-based intervention and group-based CBT intervention.

**Trial registration:**

The trial has been registered at the American Economic Association’s registry for randomized controlled trials. RCT ID: AEARCTR-0009551. Registered 2022–07-04.

## Introduction

The majority of mental disorders start before the age of 14, and they are frequently accompanied by general psychosocial disturbances that could lead to any major mental disorder. For people between the ages of 0 and 25, these disturbances are responsible for 45% of all diseases worldwide. Despite some initiatives to promote the provision of services for youth, the majority of the demands for mental health care during this critical time remain unmet. Anxiety and mood disorders, in particular, are the main cause of illness in people under the age of 25, accounting for 45% of the global disease burden (Colizzi et al., 2020). For a country with a population of 200 million, Pakistan has one of the worst mental health indicators and fewer than 500 psychiatrists. More than 90% of those with common mental disorders in Pakistan go untreated due to the severe lack of mental health professionals in the country (Sikander, 2020). The availability of trained professionals, financial resources, and patient care in the community are all lacking, even though mental health-related problems are on the rise, making access to the mental health care system still limited and dubious. Another reason why the facilities offered are not used to their full potential is the stigma associated with psychiatric labeling in society and a pervasive misconception (Khalily, 2011).

Childhood and adolescence are the critical developmental years for anxiety symptoms and syndromes, which can range from transient mild symptoms to severe anxiety disorders. Numerous studies’ findings have shown that the first or any anxiety problems are most likely to appear in children (Beesdo et al., 2009). Globally, 1 in 7 (47%) individuals between the ages of 10 and 19 will experience a mental health issue that may not receive treatment. In these age groups, emotional issues like anxiety and related disorders are very common. A total of 3.6% of children aged 10 to 14 and 4.6% of teenagers aged 15 to 19 are predicted to experience anxiety of some kind. These problems can significantly impact children’s school attendance, schoolwork, and general performance (WHO, 2022).

Anxiety can have several causes, including heredity, early life experiences, the surrounding environment, interpersonal dynamics, and other stresses. Consequences include a lack of social skills and the inability to cope with stress, fewer friendships, less pride in oneself, and less output at work, among other things (Neil & Christensen, 2009). Peer pressure, high expectations to succeed, family pressure, bullying, excessive social media use, etc. are all factors that contribute to anxiety among teenagers, as stated by McCarthy (2022). However, these conditions can be effectively treated, and relapses can be prevented if identified and addressed early with the help of trained professionals. When we talk about recovery, we do not just mean that symptoms disappear, but that people can manage them independently using learned coping mechanisms.

According to many studies, children’s mental health has been shown to improve with the help of school-based interventions (Weare & Nind, 2011; Saelid et al., 2022). Since children spend a large portion of their waking hours at school, authorities have prioritized educational institutions to prevent mental health problems and to intervene early when they do arise (Anderson et al., 2018). Both primary and early intervention can come from this area. Those who are experiencing the first episode of a mental health disorder or are just beginning to show signs of a mental health issue are prime candidates for early intervention services. Primary prevention, on the other hand, involves actions taken well before the emergence of a mental health issue. Research has also shown that the most effective and cost-effective interventions in lower-middle-income countries (LMICs) are those that combine pharmacological treatments, cognitive behavior therapy (CBT), and interpersonal psychotherapy or variants (Freeman, 2016).

Among school-aged children in Pakistan, Khalid et al. (2022) conducted a feasibility study of living life to the fullest (LLTTF), a CBT-based guided self-help program for depression, anxiety, and social functioning. A lower rate of anxiety and depression was found in participants who reported higher levels of satisfaction. The intervention also improved participants’ ability to perform daily tasks and socially adapt over time. CBT is based on the idea that changing one’s outlook on life can profoundly affect one’s mental health.

Overall, anxiety can be managed through psychotherapy, medication, or a combination of the two, depending on the nature and severity of the disorder. Although other techniques like hypnosis, biofeedback, autogenic training and acquiring new coping mechanisms are available, CBT proves to be beneficial for individuals of varying ages and backgrounds (Bandelow et al., 2017).

Due to stigmatization, there is a lack of assessment and research along with documentation of accurate mental health-related data. Additionally, due to a lack of awareness, evaluation, prevention, and interventional programs related to being implemented among adolescents in Pakistan, there is a rise in mental health issues in earlier years of life. It calls for the critical need for evidence-based intervention to promptly address the risk factors starting at the school level, which can help educate children along with teachers and parents and provide a platform for early diagnosis, treatment, and learning of healthy coping mechanisms. Schools are a powerful medium that can effectively stimulate learned information and promote mental health literacy. Hence, the current study focused on reducing this gap by carrying out a feasibility trial to assess the usefulness of the group-based therapeutic intervention at the indigenous level in a school setting among adolescents in Pakistan.

## Method

### Research design

This study was a pre-post design, two-arm, single-blinded, feasibility, randomized controlled trial. The evaluation was done at baseline (pre-assessment), endpoint (post-assessment), and follow-up after 4 weeks.

### Sample

Through the purposive sampling technique, 211 students were screened (107 male and 104 female) from four schools in Rawalpindi and were recruited for baseline assessment. The participants comprised secondary school children aged between 14 and 18 years. A total sample of 34 (17 in each arm) secondary school children with elevated scores of anxieties was then included in the trial. Since it was feasibility research, no power calculations were performed. However, it was ensured that the sample would be sufficient to predict the acceptance, uptake of the intervention, recruitment, and retention rates of participants for a future definitive trial.

### Trial design

**Fig. 1:**
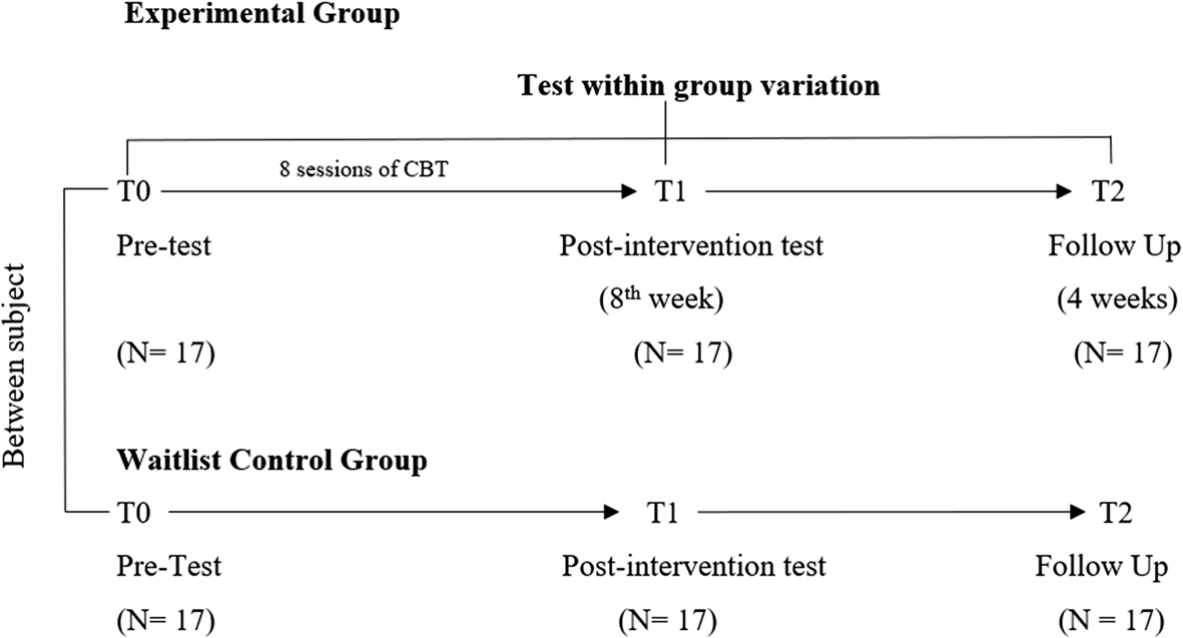
Trial design

As Fig. 1 shows, a two-arm, single-blinded, feasibility randomized controlled trial (RCT) was conducted comparing WLCG with the CBT treatment group. All the potential participants were first screened, and those who met the eligibility criteria and gave consent to participate were invited to complete a pre-testing session (T-0). Pre-testing was done to confirm their levels of anxiety by using an additional scale. After testing, participants were randomly allocated to one of the two groups: WLCG and CBT group. Participants in the CBT group completed eight sessions of CBT treatment and underwent a post-testing session (T-1) evaluating the same outcomes as the pretest to observe any differences, whereas the participants in the WLCG group also received CBT treatment after the trial was completed to comply with ethical considerations.

### Randomization

Participants were randomly assigned (1:1) to the experimental group and wait-list control group, by using a computer-generated randomization schedule supervised by an external independent, statistician ensuring strict concealment of allocation.

### Intervention group

CBT is a type of talking intervention that assists you in managing your issues by modifying your thoughts and behaviors (Chand et al., 2023). It is a type of psychological care that is useful for a variety of issues, including depression, anxiety disorders, alcohol and drug use issues, and serious mental diseases. The effectiveness of CBT has been compared to that of psychiatric drugs and other types of psychological therapy (American Psychological Association, 2022).

For the present study, group CBT-based intervention was delivered to students in class during their school hours. The intervention was adapted from the Transforming Research into Action to Improve the Lives of Students (TRAILS) group intervention for anxiety. By offering teachers, school staff, and students training, resources, and implementation assistance, TRAILS has been working to expand the provision of research-based mental health services in schools since 2013.

Participants in the CBT group completed eight sessions of CBT treatment and underwent a post-testing session (T-1) evaluating the same outcomes as the pre-test to observe any differences. For the current study, the intervention was adapted from the TRAILS group intervention for anxiety. The program consisted of eight weekly sessions, each lasting for 50 min. There was an opening session, a session on reorganizing thoughts, followed by understanding emotions, a session on problem-solving and relaxation, and a closing session with a review of the lessons/techniques learned and future planning to prevent relapse. In addition, small group of interactive exercises, worksheets, and homework was also done. Additionally, parents were also indirectly part of the study, where a parent tip sheet was sent to them to psycho-educate them.

### Wait-list control group

At the end of the trial, the participants in the wait-list-control group (WLCG) received a single-session treatment. They were also guided to believe that they would be supported if they were willing to come for therapy/intervention.

### Eligibility

#### Inclusion criteria

All potential participants, male and female secondary school children aged 14 to 18, who signed the written informed consent to participate in the trial were included. Written informed consent was obtained from these children’s parents and legal guardians before the trial began. Upon baseline assessment, children with elevated scores of anxieties (score range: 13–22) were included in the study.

### Measures

#### Behavioural and Emotional Screening System Student Form (BESS-SF)

BASC-3 Behavioural and Emotional Screening System Student Form (BESS-SF) was developed by Kamphaus and Reynolds and published in 2015. The BESS screening tool is designed for schools, mental health clinics, researchers, and communities to help screen of various emotional and behavioral problems that can later lead to adjustment problems. It comprises three main forms: parent, teacher, and student. However, for the current study, only the student form Behavioural and Emotional Screening System Student Form (BESS-SF) for children was used. It comprises 28 items in total, and a 4-point Likert scale is used to rate each item where 1—never, 2—sometimes, 3—often, and 4—almost always. The form consists of 3 subindexes: the Internalizing Risk Index (IRI) (10 items), the Self-Regulation Risk Index (6 items), and the Personal Adjustment Risk Index (8 items).

The remaining 4 items belong to the F-index, the additional behavioral and emotional risk index. The form can be completed in approximately 5 min without needing specialized training. The internal consistency coefficients for the entire Behavioural and Emotional Screening System Student Form (BESS-SF) scale were found to be ranging from adequate to strong, BESS-SF *α* = 0.90, internalizing issues *α* = 0.84, self-regulation *α* = 0.76, and personal adjustment *α* = 0.79 (Dever & Gaier, 2020).

#### The Beck Anxiety Inventory for Youth (BAI-Y II)

The Beck Anxiety Inventory for Youth (BAI-Y II), developed by Beck et al. (2005), is a self-report tool for children and adolescents aged 7 to 18 and is frequently used in academic and clinical contexts. It comprised 20 items, and 4-point Likert scale was used to rate each of them where 0 = never, 1 = sometimes, 2 = often, and 3 = Always. The Beck Anxiety Inventory for Youth (BAI-Y II) measures the physiological signs of anxiety and the specific worries that children and adolescents have related to their success in school, the future, other people’s reactions, reactions to them, fears, etc. The internal consistency was found to be *α* = 0.84 (Beck et al., 2005).

### Ethical consideration

#### Consent from institution

The research project was reviewed by the Ethical Review Board (IRB) of Foundation University Islamabad, and permission was granted to carry it out.

#### Consent from schools

Targeted schools were approached and informed about the study details. Written informed consent was obtained from them regarding voluntarily permitting to carry out the study trial on their premises.

#### Consent from parents and students

Before the trial began, written informed consent was obtained from both the participants and their parents: it was ensured that their participation was voluntary and only then were they included in the trial.

#### Trial registry and training

The trial has been registered at the American Economic Association’s registry for randomized controlled trials [RCT ID: AEARCTR-0009551. Moreover, the researcher has completed a mandatory certified training course before carrying out an RCT by the National Institute on Drug Abuse (NIDA) in collaboration with the Centre for Clinical Trials (CCT) and Clinical Trials Network (CTN) to comply with ethical standards.

### Procedure

The current study was part of a project under the Higher Education Commission, for which the feasibility of CBT intervention in a school setting was assessed. A two-arm, single-blinded, feasibility randomized controlled trial (RCT) compared WLCG with the CBT treatment group. All the potential participants were initially screened, and those who met the eligibility criteria and gave consent to participate were invited to complete a pre-testing session (T-0). After testing, participants were randomly allocated to one of the two groups: WLCG and CBT group. A sample of 34 students were recruited to become part of the trial and was given CBT-based intervention. Participants in the CBT group completed eight sessions of CBT treatment and underwent a post-testing session (T-1) evaluating the same outcomes as the pretest to observe any differences. CBT is the most effective mental illness intervention that Beck developed in the 1960s. It is a complete set of clinical intervention elements that are behavioral, cognitive, and emotional. For the current study, the intervention was adapted from the TRAILS group intervention for anxiety. The program consisted of eight weekly sessions, each lasting for 50 min. There was an opening session, a session on reorganizing thoughts, followed by understanding emotions, a session on problem-solving, relaxation, and a closing session with a review of the lessons/techniques learned and future planning to prevent relapse. In addition to a small group of interactive exercises through worksheets and homework, each session was taught with a PowerPoint presentation. Additionally, parents were also indirectly part of the study, where a parent tip sheet was sent to them to psycho-educate them. Moreover, the research team also conducted teacher training.

### Data analysis

For the present study, all statistical analyses were conducted using SPSS 20.0 version software (SPSS, Inc., Chicago, IL, USA). A two-way factorial ANOVA was done for the primary endpoint analysis. The mean difference between two treatment arms, three time points (pre-, post, and follow-up), together with its 95% confidence interval (CI), was derived from the multivariate analysis. Effect sizes were estimated using the partial eta-squared (η^2^p) with a significance level of < . 05.

### CONSORT

**Fig. 2:**
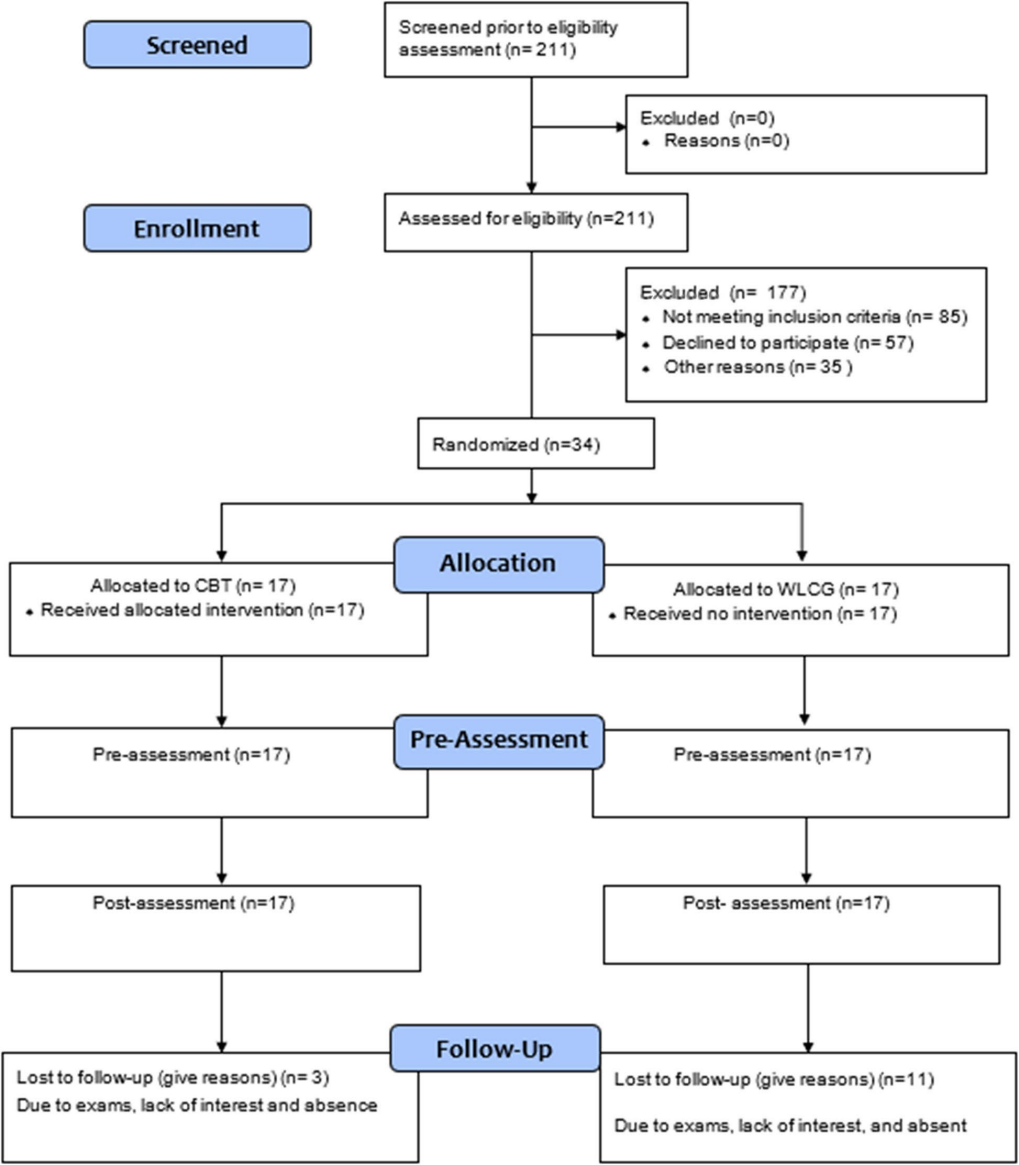
CONSORT

Consolidated Standards of Reporting Trials (CONSORT) was developed to assist researchers in reporting their randomized controlled trials (RCTs). The results of RCTs are most valuable when authors correctly, completely, and honestly describe their procedures and findings (Grant et al., 2018). Hence, the Consolidated Standards of Reporting Trials (CONSORT) was used to maintain transparency in reporting and maximize the credibility of the present study. In total, 34 participants gave their consent and were randomized into one of the two groups: WLCG (*n* = 17) and CBT (*n* = 17). The flow chart of participants is given in Fig. 2.

## Results

To assess the trial’s effectivity at three levels, sample demographic characteristics, internal consistencies, and repeated-measure ANOVA were used.

Table 1 depicts the descriptive mean, standard deviation, and alpha reliability of the primary study variables. Descriptive statistics were obtained to present the data distribution and the average results for the study subjects on the study variables. The magnitude of alpha coefficients for the Behavioural and Emotional Screening System Student Form was 0.86, showing good internal consistency, while that of BAI-Y was 0.70.

**Table 1:** Descriptive statistics and psychometric properties of BESS-SF and BAI-Y main scales and subscales (*N* = 211)

Variable	*k*	*α*	*M*	*SD*	Range	
					Potential	Actual
BESS-SF	28	0.86	31.16	12.16	28–112	3–67
IRI	9	0.77	12.51	5.51	9–36	2–30
SRI	6	0.41	6.64	2.57	6–24	.0–14
PARI	8	0.75	6.4	4.2	8–32	.0–19
BAIY	20	0.70	35.19	6.88	20–80	17–50

*K* Number of items, *BESS-SF* Behavioural and emotional screening system student form, *IRI* Internalizing risk index, *SRI* Self-regulation index, *PARI* Personal adjustment risk index, *BAIY* Beck anxiety inventory-youth

Table 2 revealed that around 61% of the participants were male and 38% were females with mean age of 15 (*M* = 15, *SD* = 0.73). Moreover, 55% were from the 9th grade, and 44% belonged to the10th grade. A total of 67% were from the nuclear family system and 32% from the joint family system.

**Table 2:** Demographic characteristics of participants (*N* = 34)

Baseline characteristics	Category	*f (%)*
Class		
	9th	19 (55.9)
	10th	15 (44.1)
Gender		
	Male	21 (61.8)
	Female	13 (38.2)
School		
	School 1	6 (17.6)
	School 2	16 (47.1)
	School 3	6 (17.6)
	School 4	6 (17.6)
Family system		
	Nuclear	23 (67.6)
	Joint	11 (32.4)

Table 3 shows the relationship among study variables. Results depict that internalizing risk factor index (*r* = 0.86, *p* =  < 0.01), self-regulation index (*r* = 0.66, *p* =  < 0.01), and personal adjustment risk (*r* = 0.82, *p* =  < 0.01) adjustment all have a significantly positive relationship with the Behavior Assessment Scale for children. Moreover, the Beck Anxiety Inventory for Youth has a nonsignificant negative relationship with the personal adjustment risk index *r* =  − 0.30. Similarly, the Beck Anxiety Inventory for Youth also has a significantly positive relationship with the internalizing risk factor index (*r* = 0.37, *p* =  < 0.05).

**Table 3:** Relationship between BASC, IRI, SRI, PARI, and BAI-Y (*N* = 34)

	1	2	3	4	5
1.BESS-SF	-				
2.IRI	0.86^b^	-			
3.SRI	0.66^b^	0.48^b^	-		
4.PARI	0.82^b^	0.55^b^	0.41^b^	-	
5.BAIY	.08	0.37^a^	0.23	–0.30	-

*BASC* Behavior assessment scale for children, *IRI* Internalizing risk factors index, *SRI* Self-regulation index, *PARI* Personal adjustment risk index, *BAIY* Beck anxiety inventory for youth

^a^*p* < .05

^b^*p* < .01

Table 4 shows a significant effect for the IRI in experimental group (*F* = 8.45, *p* = 0.009, ηp2 = 0.32), whereas the wait-list control group participants showed no significant improvement in pre (*M* = 16.52, *SD* = 4.09), post (*M* = 16.58, *SD* = 4.66), and follow-up (*M* = 15.33, *SD* = 3.55). Participants in the experimental group (CBT) showed a significant improvement in IRI in postcondition (*M* = 7.52, *SD* = 2.34) compared to pre-condition (*M* = 21.11, *SD* = 3.68). Contrarily, in follow-up (*M* = 8.78, *SD* = 1.18), a slight increase in IRI was observed as no treatment was provided. Similarly, there was significant decrease in SRI (*F* = 5.80, *p* = 0.000, *ηp2* = 0.24), PARI (*F* = 20.08, *p* = 0.000, *ηp2* = 0.52), and BAI-Y (*F* = 30.44, *p* = 0.000, *ηp2* = 0.62).

**Table 4:** Mean differences between the wait-list control group and experimental group on BESS-SF and BAI-Y (*N* = 34)

	WLCG (*n* = 17)		CBT (*n* = 17)		Group			Time			Group × time			95% *CI*	
	*M*	*SD*	*M*	*SD*	*F*	*p*	ηp2	*F*	*p*	ηp2	*F*	*p*	ηp2	LL	UL
**IRI**															
Pre	16.52	4.09	21.11	3.68	8.45	.009*	0.32	21.74	.000*	0.54	21.74	.000*	0.54	19.1	23.0
Post	16.58	4.66	7.52	2.34											
FU	15.33	3.55	8.78	1.18											
**SRI**															
Pre	8.05	3.27	9.78	2.66	5.80	.02*	0.244	19.84	.000*	0.52	17.50	.001*	0.49	8.0	10.66
Post	8.66	3.36	6.92	3.64											
FU	15.33	2.06	10.00	1.03											
**PARI**															
Pre	16.83	3.48	11.00	3.63	20.08	.000*	0.52	25.02	.000*	0.58	11.90	.003*	0.39	8.67	10.32
Post	18.66	4.27	11.42	3.27											
FU	8.66	1.03	9.50	1.60											
**BAIY**															
Pre	33.29	4.93	33.17	8.95	30.44	.000*	0.62	28.23	.000*	0.61	3.53	.07	0.16	11.83	19.88
Post	33.17	8.95	11.94	2.96											
FU	23.66	10.76	15.85	5.15											

*IRI* Internalizing risk factors index, *SRI* Self-regulation index, *PARI* Personal adjustment risk index, *BAI-Y* Beck anxiety inventory-youth, *p< .05

## Discussion

Anxiety among students is less often identified and treated in educational settings than other mental health issues. According to a recent study, anxiety symptoms were present in 20% of adolescents where untreated mental health issues had both short- and long-term effects on one’s health and well-being (Adams et al., 2022). Similarly, school environment presents a one-of-a-kind opportunity to broaden students’ access to anxiety-based supports and therapies. However, very little is known about the school-based providers regarding the anxiety of students and the school-based interventions and supports that are available. Despite the many benefits of providing mental health support and services in the school environment, very little to no intervention-based studies have been carried out in Pakistan. The present study aimed to seek a feasibility evaluation of a group-based intervention for anxiety using CBT to fill this gap.

CBT has proven to be an effective treatment for childhood anxiety and related disorders (Kreuze et al., 2018). For instance, a review of 50 years’ worth of outcome studies on children and teenagers with anxiety disorders demonstrated good evidence in favor of symptom improvement in CBT, with significant pre- to posttreatment effect sizes (Levy et al., 2022). With a 59% posttreatment remission rate, CBT is a successful treatment for young people with anxiety and associated illnesses, where reviews and meta-analyses’ findings show that treatment benefits—at least in terms of symptom improvement—are maintained throughout long-term follow-up (James et al., 2013).

The present study’s findings depict that BASC, which comprises three sub-scales self-regulation index, personal adjustment index, and internalizing risk index (IRI), has a significant positive relationship. Furthermore, anxiety (BAIY) and IRI are also significantly positively correlated. This is why results revealed that CBT also had a positive impact on self-regulation as well as personal adjustment of participants. Studies have also proved how anxiety and emotional regulation are related, which means that one has an impact on the other as well. Similarly, worried children may show actual social skill difficulties (Dodd et al., 2011). Emotional self-regulation is the extrinsic and intrinsic systems responsible for monitoring, analyzing, and altering emotional reactions, and it depends on adapting reactions to situational demands. While this skill develops throughout life, children have their primary regulation techniques by age 7. Help-seeking, avoidance, attentional redirection, suppression, and problem-solving are few methods. Children who fail to regulate their emotions are at risk for internalizing symptoms. Children who see a situation as unmanageable or have a strong undesirable feeling to regulate their emotion have a higher risk of internalizing problems. If emotion regulation is ineffective, undesired feelings may proliferate, leading to more psychological distress and ineffective emotion regulation that can further lead to anxiety or depression (Loevaas et al., 2018). In support of these studies, a longitudinal investigation also indicated that poor emotion regulation skills predicted internalizing symptoms in children (Kim-Spoon et al., 2012; Suveg & Zeman, 2004; Zeman et al., 2002).

A mixed ANOVA analysis (Table 2) was done to measure the effectiveness of the therapy at three levels: pre, post, and follow-up. The trial result indicated that the level of anxiety among school children in T1 decreased significantly more than in T0 while showing a slight increase in T3, i.e., at follow-up. The main goal of psychological therapy was to persuade the participants to adopt healthy life principles and how to tolerate unpleasant experiences, including thoughts, feelings, and unavoidable bodily sensation. The therapy focused on altering cognitions to alter emotions and behaviors. In CBT, the facilitator introduced adaptive coping skills and gave opportunities for implementing and rehearsing strategies to develop a sense of mastery over suffering and manage tough situations caused by anxiety and anxiety symptoms. Various studies support that key components for CBT-based intervention like challenging maladaptive thoughts and behaviors and reassembling them in a more truthful and balanced manner, relaxation, relapse prevention, exposure, and psycho-education were useful in reducing anxiety among children. The effectiveness and usefulness of exposure therapy for anxiety disorders have been thoroughly proven and are regarded as the treatment of choice for many types of pathological anxiety (Clark, 2013; Kaczkurkin & Foa, 2015; Mukund & Jena, 2022; Otte, 2011; Zaboski, 2020). Similarly, identifying individual and environmental factors and educating participants about future relapse are equally essential (Bowen et al., 2014).

To conclude, the intervention positively impacted young adults, reducing anxiety as the primary outcome. A recent study by Haugland and peers in 2020 also concluded that anxiety symptoms can be effectively prevented by focused school-based interventions with reports of young adult anxiety showing positive effects due to CBT (Haugland et al., 2020).

### Limitations

The current study was a feasibility trial, and although anxiety was reduced and students were educated about their mental health, the sample size was still very small because it was collected from only four schools. In addition to expanding the sample size to 57, more studies need to be done to determine what additional factors, such as parenting styles and coping mechanisms, contribute most to the success of a given treatment over the long run.

The short duration of the study and the fact that the intervention was only followed up 3 weeks later preclude any conclusions being drawn about the long-term consequences of the treatment.

The absence of a validation study for the Behavioural and Emotional Screening System Student Form (BESS-SF) in Pakistan introduces significant limitations, potentially leading to cultural misinterpretations and inaccurate measurements. Consequently, the findings may need to be more generalizable to the broader Pakistani population, underscoring the need for local validation efforts.

The lack of a validation study for the Beck Anxiety Inventory for Youth (BAI-Y II) in Pakistan introduces significant limitations. Using the BAI-Y II without a locally validated version may lead to cultural misinterpretations and inaccuracies in measuring anxiety, potentially biased the results, and limiting their reliability and generalizability to the Pakistani youth population.

## Conclusion

This feasibility study evaluated the efficacy of a group CBT-based intervention for anxiety among school-aged children, determining that anxiety levels significantly decreased after eight therapeutic sessions conducted over 2 months. Additionally, the intervention reduced aggression and improved social relationships among the children. The psycho-education component equipped children with knowledge about relapse prevention and guidance on when and how to seek future help, highlighting the intervention’s comprehensive approach and its potential importance and relevance for addressing anxiety in school-aged populations.

## Data Availability

The datasets used and/or analyzed during the current study are available from the corresponding author upon reasonable request.

## Abbreviations

CBT: Cognitive behavioral therapy
RCT: Randomized controlled trial
LMICS: Lower-middle-income countries
CONSORT: Consolidated Standards of Reporting Trials
TRAILS: Transforming Research into to Improve the Lives of Students
